# Supplementary material for: Emotionotopy in the human right temporo-parietal cortex
Source: Nat Commun. 2019 Dec 5;10:5568. doi: 10.1038/s41467-019-13599-z (PMC6895053; doi:10.1038/s41467-019-13599-z)
Supplement: Supplementary file 3 — Reporting Summary [file 41467_2019_13599_MOESM3_ESM.pdf]

nature research

Corresponding author(s): Luca Cecchetti

Last updated by author(s): Oct 10, 2019

Reporting Summary

Nature Research wishes to improve the reproducibility of the work that we publish. This form provides structure for consistency and transparency in reporting. For further information on Nature Research policies, see Authors & Referees and the Editorial Policy Checklist.

Statistics

For all statistical analyses, confirm that the following items are present in the figure legend, table legend, main text, or Methods section.

n/a	Confirmed	
☐	☒	The exact sample size (*n*) for each experimental group/condition, given as a discrete number and unit of measurement
☐	☒	A statement on whether measurements were taken from distinct samples or whether the same sample was measured repeatedly
☐	☒	The statistical test(s) used AND whether they are one- or two-sided<br>*Only common tests should be described solely by name; describe more complex techniques in the Methods section.*
☐	☒	A description of all covariates tested
☐	☒	A description of any assumptions or corrections, such as tests of normality and adjustment for multiple comparisons
☐	☒	A full description of the statistical parameters including central tendency (e.g. means) or other basic estimates (e.g. regression coefficient) AND variation (e.g. standard deviation) or associated estimates of uncertainty (e.g. confidence intervals)
☐	☒	For null hypothesis testing, the test statistic (e.g. *F*, *t*, *r*) with confidence intervals, effect sizes, degrees of freedom and *P* value noted<br>*Give P values as exact values whenever suitable.*
☒	☐	For Bayesian analysis, information on the choice of priors and Markov chain Monte Carlo settings
☒	☐	For hierarchical and complex designs, identification of the appropriate level for tests and full reporting of outcomes
☐	☒	Estimates of effect sizes (e.g. Cohen's *d*, Pearson's *r*), indicating how they were calculated

Our web collection on statistics for biologists contains articles on many of the points above.

Software and code

Policy information about availability of computer code

Data collection	MATLAB 2016b running Psychtoolbox v 3.0.14
Data analysis	ANTs, AFNI v17.2.00, MATLAB 2016b

For manuscripts utilizing custom algorithms or software that are central to the research but not yet described in published literature, software must be made available to editors/reviewers. We strongly encourage code deposition in a community repository (e.g. GitHub). See the Nature Research guidelines for submitting code & software for further information.

Data

Policy information about availability of data

All manuscripts must include a data availability statement. This statement should provide the following information, where applicable:

- Accession codes, unique identifiers, or web links for publicly available datasets
- A list of figures that have associated raw data
- A description of any restrictions on data availability

The datasets generated and analysed during the current study are publicly available in the studyforrest.org (http://psydata.ovgu.de/studyforrest/phase2/) repository and at OpenScience foundation (https://osf.io/tzpdf).

Field-specific reporting

Please select the one below that is the best fit for your research. If you are not sure, read the appropriate sections before making your selection.

☒ Life sciences ☐ Behavioural & social sciences ☐ Ecological, evolutionary & environmental sciences

Life sciences study design

All studies must disclose on these points even when the disclosure is negative.

Sample size	Twenty-seven subjects participated in our study. The available dataset that we used (Hanke et al., 2016, 10.1038/sdata.2016.92) was composed by 15 subjects, who underwent 2 hours of fMRI scanning (3,599 timepoints) each. The sample size (n=12) for the behavioral experiment was matched to the fMRI one and the same amount of data (2h acquisition of emotion ratings) was acquired for each of the subjects included in the behavioral experiment.
Data exclusions	One subject of the fMRI experiment was excluded since recordings of physiological parameters were not available. Thus, the final fMRI sample consisted of 14 subjects.
Replication	The gradient-like organization of TPJ was tested both at group-level and in the single subject data. Raw fMRI and behavioral data, as well as the code employed in the analyses, are publicly available.
Randomization	Both groups (behavioral and fMRI data) were matched for age and gender and were randomly sampled from the general population.
Blinding	Blinding was not relevant in our design.

Reporting for specific materials, systems and methods

We require information from authors about some types of materials, experimental systems and methods used in many studies. Here, indicate whether each material, system or method listed is relevant to your study. If you are not sure if a list item applies to your research, read the appropriate section before selecting a response.

Materials & experimental systems

n/a	Involved in the study	
☒	☐	Antibodies
☒	☐	Eukaryotic cell lines
☒	☐	Palaeontology
☒	☐	Animals and other organisms
☐	☒	Human research participants
☒	☐	Clinical data

Methods

n/a	Involved in the study	
☒	☐	ChIP-seq
☒	☐	Flow cytometry
☐	☒	MRI-based neuroimaging

Human research participants

Policy information about studies involving human research participants

Population characteristics	In the fMRI study, 6 females and 8 males (mean age 29.4, min 20, max 40 years) were acquired and analysed. In the behavioral study we enrolled 5 females and 7 males (mean age 26.6, min 24, max 34 years).
Recruitment	Subjects responded to a bulletin posting or were recruited by word of mouth.
Ethics oversight	Area Vasta Nord Ovest Ethics Committee Protocol N°1485/2017

Note that full information on the approval of the study protocol must also be provided in the manuscript.

Magnetic resonance imaging

Experimental design

Design type	Continuous naturalistic stimulation (audiovisual version of the Forrest Gump movie).
Design specifications	8 runs of fMRI data were acquired in two sessions for a total of 3,599 brain volumes.
Behavioral performance measures	Subjects were instructed to inhibit any movement and simply enjoy the Forrest Gump movie.

Acquisition

Imaging type(s)	Functional.
Field strength	3T.
Sequence & imaging parameters	Gradient-echo EPI, TR 2s, TE 30ms, FA 90°, 3mm ISO, FoV 240mm, slice thickness 3.0 mm.
Area of acquisition	Whole brain.

Diffusion MRI ☐ Used ☒ Not used

Preprocessing

Preprocessing software	ANTs and AFNI v17.2.00.
Normalization	Non-linear normalization.
Normalization template	MNI152.
Noise and artifact removal	Motion and physiological parameters were used as nuisance regressors.
Volume censoring	No censoring was applied. However, framewise displacement was included into the model to mitigate spurious activity related to head movement.

Statistical modeling & inference

Model type and settings	Voxelwise encoding analysis, using behavioral ratings as predictors and fMRI activity as the dependent variable in a multiple regression procedure.
Effect(s) tested	We tested the association (R^2) between behavioral ratings and fMRI activity. We assessed the statistical significance of the R^2 using a permutation approach, by generating 10,000 null models having the same spectral density and temporal autocorrelation of our predictors (Iterative Amplitude Adjusted Fourier Transform).

Specify type of analysis: ☒ Whole brain ☐ ROI-based ☐ Both

Statistic type for inference (See Eklund et al. 2016)	Voxelwise
Correction	FDR correction, q<0.01.

Models & analysis

n/a	Involved in the study
☒	☐ Functional and/or effective connectivity
☒	☐ Graph analysis
☐	☒ Multivariate modeling or predictive analysis

Multivariate modeling and predictive analysis	Independent predictors comprised our behavioral ratings of perceived intensity of basic emotions. fMRI activity was the dependent variable. The gradient-like organization of TPJ was tested by measuring the association (i.e., Spearman's rho) between two dissimilarity matrices: one using the Euclidean distance of voxel coordinates (anatomical distance), and the other one using the Euclidean distance of coefficients related to the fitting of a specific model (functional distance). The statistical significance was measured by generating a null distribution which maintained the same spatial autocorrelation structure of TPJ.
